# Connections between cross-tissue and intra-tissue biomarkers of aging biology in older adults

**DOI:** 10.1186/s43682-023-00022-4

**Published:** 2023-10-12

**Authors:** R. Waziry, Y. Gu, O. Williams, S. Hägg

**Affiliations:** 1 Department of Neurology, Columbia University Irving Medical Center, Vagelos College of Physicians and Surgeons, Columbia University, New York, NY 10032, USA; 2 The Taub Institute for Research in Alzheimer’s Disease and the Aging Brain, Columbia University, New York, NY, USA; 3 G.H. Sergievsky Center, Vagelos College of Physicians and Surgeons, Columbia University, New York, New York, USA; 4 The Department of Epidemiology, Joseph P. Mailman School of Public Health, Columbia University, New York, NY, USA; 5 Department of Medical Epidemiology and Biostatistics, Karolinska Institutet, Solna, Sweden

## Abstract

**Background:**

Saliva measures are generally more accessible than blood, especially in vulnerable populations. However, connections between aging biology biomarkers in different body tissues remain unknown.

**Methods:**

The present study included individuals (*N* = 2406) who consented for saliva and blood draw in the Health and Retirement Telomere length study in 2008 and the Venous blood study in 2016 who had complete data for both tissues. We assessed biological aging based on telomere length in saliva and DNA methylation and physiology measures in blood. DNA methylation clocks combine information from CpGs to produce the aging measures representative of epigenetic aging in humans. We analyzed DNA methylation clocks proposed by Horvath (353 CpG sites), Hannum (71 CpG sites), Levine or PhenoAge, (513 CpG sites), GrimAge, (epigenetic surrogate markers for select plasma proteins), Horvath skin and blood (391 CpG sites), Lin (99 CpG sites), Weidner (3 CpG sites), and VidalBralo (8 CpG sites). Physiology measures (referred to as phenotypic age) included albumin, creatinine, glucose, [log] C-reactive protein, lymphocyte percent, mean cell volume, red blood cell distribution width, alkaline phosphatase, and white blood cell count. The phenotypic age algorithm is based on parametrization of Gompertz proportional hazard models. Average telomere length was assayed using quantitative PCR (qPCR) by comparing the telomere sequence copy number in each patient’s sample (T) to a single-copy gene copy number (S). The resulting T/S ratio was proportional to telomere length, mean. Within individual, relationships between aging biology measures in blood and saliva and variations according to sex were assessed.

**Results:**

Saliva-based telomere length showed inverse associations with both physiology-based and DNA methylation-based aging biology biomarkers in blood. Longer saliva-based telomere length was associated with 1 to 4 years slower biological aging based on blood-based biomarkers with the highest magnitude being Weidner (*β* = − 3.97, *P* = 0.005), GrimAge (*β* = − 3.33, *P* < 0.001), and Lin (*β* = − 3.45, *P* = 0.008) biomarkers of DNA methylation.

**Conclusions:**

There are strong connections between aging biology biomarkers in saliva and blood in older adults. Changes in telomere length vary with changes in DNA methylation and physiology biomarkers of aging biology. We observed variations in the relationship between each body system represented by physiology biomarkers and biological aging, particularly at the DNA methylation level. These observations provide novel opportunities for integration of both blood-based and saliva-based biomarkers in clinical care of vulnerable and clinically difficult to reach populations where either or both tissues would be accessible for clinical monitoring purposes.

## Introduction

Aging is hypothesized to be a key driver of major age-related pathologies and vascular disease [1–3]. Measures of aging biology have been proposed as a proxy to the global aging status of an individual [4]. Different tissues are commonly used, including saliva and blood derivatives, to measure biological aging. Blood tissues used include venous versus capillary and whole blood versus point of care and other blood products such as plasma [5]. Saliva is considered one of the most accessible body tissues and have been effectively used in clinically difficult populations, among whom obtaining blood access is not feasible [6]. Clinical uses of saliva include monitoring drug doses of cardiovascular and neurological disorders such as diabetes mellitus, multiple sclerosis, and epilepsy [6]. It has proven economic efficacy due to its easier access and the need for less materials and time to obtain, ship, and store samples [7, 8].

There are variations in concentration and abundance of molecular analytes between saliva and blood [9]. These established differences would prompt the use of either tissue according to the targeted investigation, age group, or endpoint being assessed [10]. Differences in abundance may therefore directly affect the precision of what is measured and may translate in differences in predicting risk of complications [10–12]. Similarly, differences between blood and saliva include variations according to age, sex, type and size of salivary gland, blood type, and physiological status, which are more likely to influence salivary tissue content [13, 14].

The precise differences between those measures would provide opportunities for more effective diagnostic and prognostic tools in clinical medicine and would open avenues for interventions at a whole population scale as cost-effective screening and monitoring tools become available in the near future [14]. In the present study, we aimed to assess connections between cross-tissue (i.e., saliva-blood) and intra-tissue (i.e., blood-blood) aging biomarkers to evaluate whether saliva-based telomere length biomarkers of aging biology reflect changes in blood-based DNA methylation and physiology biomarkers in older adults.

## Methods

### Study population

The Health and Retirement Study (HRS) is a nationally representative longitudinal survey that recruited more than 37,000 individuals aged 50 and older in the USA. The survey has been conducted every 2 years since 1992 with a focus on issues related to changes in health and economic circumstances in aging at both the individual and population levels [15]. HRS data are linked to records from Social Security, Medicare, Veteran’s Administration, the National Death Index, and employer-provided pension plan information. Genetic ancestry in HRS is identified through PC analysis on genome-wide SNPs [15]. HRS is coordinated by the Institute for Social Research at the University of Michigan. The present study included individuals who consented for saliva and blood draw in the health and retirement telomere length study in 2008 and the venous blood study in 2016 respectively and who had complete data for both tissues (*N* = 2406) [15–17]. Individuals without data on telomere length, physiology, and DNA methylation measures were not included in the present investigation to allow for direct individual comparisons using blood-based biomarkers and saliva-based biomarkers. The included sample had similar overall age and sex distribution to the full sample with weighted mean age equals 67 and 54% females. The Health and Retirement Study is reviewed and approved by the University of Michigan’s Health Sciences IRB. The present study has been conducted in accordance with STROBE guidelines for observational studies (https://www.strobe-statement.org/).

### Biological aging biomarkers

We assessed biological aging based on (A) telomere length, (B) DNA methylation, and (C) physiology measures.

#### (A) Telomere length measurement

Telomere length data were available from 5808 HRS respondents who consented for saliva sample draw during the 2008 interview wave. Assays were performed by Telome Health (Telomere Diagnostics, http://www.telomehealth.com/). Average telomere length was assayed using quantitative PCR (qPCR) by comparing telomere sequence copy number in each patient’s sample (T) to a single-copy gene copy number (S). The resulting T/S ratio was proportional to telomere length, mean. The HRS *2008 Telomere* data set is sponsored by the National Institute on Aging (NIH NIAU01AG009740 and RC4 AG039029) and was conducted by the University of Michigan [17].

##### The venous blood substudy (VBS) and assay protocol in 2016 Health and Retirement Study

All respondents, with the exception of proxy and nursing home respondents, who completed the HRS interview in 2016 (visit 13) were asked to consent for blood draw (78.5% of participants interviewed through September 5, 2017) [16]. Physiology-based biomarkers were assessed on the whole sample of participants who consented for blood draw, while DNA methylation was assessed in a subsample randomly selected and fully representative of the whole sample [16]. Blood samples were centrifuged and shipped overnight to CLIA-certified Advanced Research and Diagnostic Laboratory at the University of Minnesota. Tube processing was done within 24 h of arrival at the lab (within 48 h of collection). All assays were done at the University of MN Advanced Research and Diagnostic Laboratory (ARDL) under the direction of Bharat Thyagarajan.

#### (B) DNA methylation

DNA methylation assays were done by Infinium Methylation EPIC BeadChip at the University of Minnesota following the manufacturer’s instruction. The minfi package in R was used in data processing, and DNA methylation measures were provided through HRS to the research community [18, 19]. Epigenetic clocks are commonly based on portions of the genome where methylation changes are related to chronological age and, more recently, health outcomes [18]. The clocks combine information from CpGs to produce the aging measure that represents an indicator of epigenetic aging [18]. DNA methylation assessment included the following clocks: Horvath [20, 21] and Hannum et al. [22], Levine et al. or PhenoAge [23], Lu et al. (referred to as GrimAge) [24], Horvath skin and blood [25], Lin [26–28], Weidner [28], and VidalBralo [29]. The Horvath epigenetic clock predicts age using 353 CpG sites in the DNA methylation profile and incorporates 51 healthy tissues and cell types [20, 21]. The Hannum et al. clock comprises 71 CpG sites selected from the Illumina 450 k array that capture changes in chronological age. It was developed in whole blood of humans at ages 19 to 101 [22]. DNAm PhenoAge based on 513 CpGs predicted phenotypic age in whole blood from the same sample [23]. GrimAge is a mortality predictor and was constructed based on surrogate markers for select plasma proteins ((adrenomedullin, β-2-microglobulin, CD56, ceruloplasmin, cystatin C, EGF fibulin-like ECM protein 1, growth differentiation factor 15, leptin, myoglobin, plasminogen activator inhibitor 1, serum paraoxonase/arylesterase 1, and tissue inhibitor metalloproteinases 1) and smoking pack-years in a two-stage procedure [24].GrimAge is suggested to have predictive ability for time to death, coronary heart disease, cancer, and age-related conditions [24]. Skin and blood was developed as a novel and highly robust DNAm age estimator (based on 391 CpGs) for human fibroblasts, keratinocytes, buccal cells, endothelial cells, lymphoblastoid cells, skin, blood, and saliva samples [25]. The clock is suggested to have high age correlations in sorted neurons, glia, brain, liver, and bone samples [25]. The skin and blood clock shares 45 CpGs with blood-based Hannum and 60 CpGs with Horvath pan-tissue clock [21, 22]. Lin was developed using a 99-CpG aging model derived in DNAm profiles of normal blood samples and trained on life expectancy [26–28].Weidner was developed based on three age-related CpGs located in the genes ITGA2B, ASPA, and PDE4C to estimate epigenetic aging in blood [28]. VidalBralo was developed in whole blood of 8 CpG sites that were selected as the most informative CpGs in a training dataset of 390 healthy subjects and validated in three datasets [29].

The chronological classification of the clocks can be summarized as follows: the first-generation clocks were developed using machine learning to predict chronological age. These clocks demonstrated two important proofs of concept; they recorded increases in clock age within individuals as they grew older [30, 31], and more advanced clock-age estimates (i.e., clock ages older than chronological age) were associated with increased mortality risk among individuals of the same chronological age [23]. Second-generation DNAm clocks were developed from analysis of mortality risk, incorporating information from DNAm prediction of physiological parameters [23, 24]. These second-generation clocks are more predictive of morbidity and mortality [32] and are proposed to have improved potential for testing impacts of interventions to slow aging [33].

#### (C) Physiology measures

Among available and validated measures of biological aging based on physiologic parameters, we opted to use physiology-based phenotypic age described in detail by Liu et al. [34]. This clock uses the following nine biomarkers assessed in blood representing the different body systems: albumin, creatinine, glucose, [log], C-reactive protein [CRP], lymphocyte percent, mean cell volume, red blood cell distribution width, alkaline phosphatase, and white blood cell count [35]. These biomarkers were selected using a Cox proportional hazard elastic net model for mortality. The phenotypic age algorithm is based on parametrization of two Gompertz proportional hazard models, one including the 10 selected variables and the other including only chronological age [34, 36].

### Statistical analysis

Summary statistics were assessed as median (IQR) for continuous variables and percentages for categorical variables. Survey weights were used in descriptive analysis [37, 38]. Correlation coefficients (Pearson) between cross-tissue (saliva-blood) and intra-tissue (blood-blood) measures were assessed for magnitude, direction, and statistical significance with correction for multiple comparison [39, 40]. We have also assessed correlations between each physiological biomarker with telomere length and DNA methylation biological aging measures. Linear regression models were conducted with DNA methylation or physiology-based biological aging measure as the dependent variable. Blood measures were used in the raw form to allow for direct comparisons. Telomere length was logged and included in the models as tertiles [41]. Tertiles were used to allow for interpretable change per unit increase in telomere length. Models were adjusted for sex, sex and telomere length interaction, and chronological age difference in years between time at saliva and blood draws. These covariates were determined a priori with emphasis on sex and chronological age for their established biological relevance and data completeness [35, 42]. Inclusion of sex and telomere length interaction term was determined a priori based on hypothesized sex-based differences in telomeres [42–45]. Sex-stratified models adjusted for chronological age difference in years between time at saliva and blood draws were also conducted. Sensitivity analysis was conducted to assess the models after exclusion of telomere length T/S ratio greater than 2.0 since greater values are more likely to be artificial in salivary samples [46]. Models were also assessed without sex and telomere length interaction term. All analyses were conducted using Stata SE V.16.0.

## Results

### Population characteristics

A total of 2406 individuals had complete data on salivary telomere length and physiology-based measurements, and 1029 had complete data on salivary telomere length, physiology-based, and DNA-based measurements. Median age for the study sample at the time when saliva was drawn was 66 years (*IQR* 59, 72), median age at time of blood draw was 74 (*IQR* 67, 80), the majority were females (60%), and African Americans comprised 11% of the sample, while the majority were Whites (77%). In terms of self-reported health, respondents who reported “very good” and “good” represented 34% and 31%, respectively. Median time difference between saliva and blood draw was 8 years (*IQR* 8, 8.3) (Table 1, Supplementary Table 1). For saliva-based telomere length, median telomere length was 1.30 (*IQR* 1.14 and 1.50). For blood-based measures of DNA methylation, median biological ages were as follows: Horvath 69.00, Hannum 57.80, Levine 60.70, skin and blood 73.54, Lin 61.60, Weidner 67.43, VidalBralo 65.15, and GrimAge 71.30. Median age for physiology-based biological aging, phenotypic age, was 74.41 (Table 1). Weighted summary for variables included is described in Supplementary Table 1.

### Connections between cross-tissue (saliva–blood) biomarkers of biological aging

There was an inverse relationship between saliva-based telomere length and all blood-based measures, such that longer telomeres reflected younger biological aging. Measures that showed statistically significant correlation coefficients included Lin (− 0.067, *P* = 0.031) and Weidner (− 0.064, *P* = 0.037) (Table 2).

**Fig. 1:**
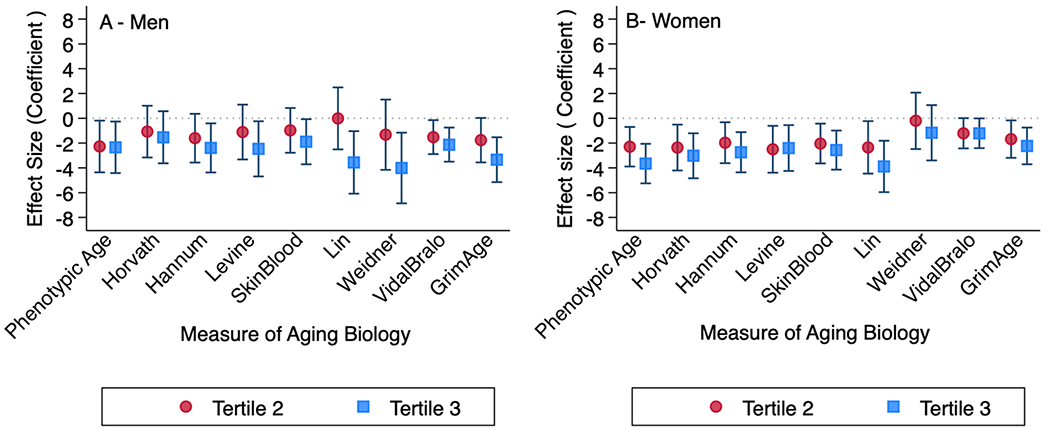
Relationship between biological age measures in blood and telomere length (tertiles)

In tertile analysis of saliva-based telomere length, compared to the lowest tertile, longer saliva-based telomere length was associated with 1 to 4 years slower biological aging based on blood-based biomarkers with the highest magnitude being Weidner (*β* = − 3.97, *P* = 0.005), GrimAge (*β* = − 3.33, *P* < 0.001), and Lin (*β* = − 3.45, *P* = 0.008) biomarkers of DNA methylation. Models were adjusted for sex, sex and telomere length interaction, and time difference between saliva and blood draw. Similar results were observed after exclusion of telomere length T/S ratio greater than 2 (Table 3, Supplementary Table 2). Models without the sex-telomere length interaction term showed highest magnitude with Lin (reference lowest tertile, *β* = − 3.69, *P* < 0.001) and phenotypic age (*β* = − 3.14, *P* < 0.001). Sex-stratified models showed variations in effect estimates across measures of biological aging in blood between males and females with every tertile increase in telomere length (Fig. 1).

Estimates varied widely across the nine physiological biomarkers with DNA methylation measures with the highest correlation observed with creatinine and lymphocyte percent (Supplementary Tables 3 and 4).

### Connections between intra-tissue (blood–blood) biomarkers of biological aging

In a secondary analysis, all blood-based biomarkers of DNA methylation showed directly proportional and strong statistically significant relationships with blood-based physiology measures of biological aging. Among blood-based DNA methylation biomarkers, GrimAge showed the strongest correlation with blood-based physiological measure, phenotypic age (correlation coefficient = 0.75, *P* < 0.001) followed by Hannum (0.68, *P* < 0.001), and Horvath skin and blood measure (0.66, *P* < 0.001) (Table 2).

## Discussion

In the present study, we found that increases in telomere length measured in saliva was reflected in younger biological age based on DNA methylation and physiology measured in blood (*P* < 0.001) 8 years later. Such that one tertile increase in telomere length in saliva translated to approximately 4 years younger biological age in blood measures, with some variations between males and females, in a representative population of older adults, despite relatively weak correlations between cross-tissue measures compared to intra-tissue measures in blood. We also observed correlations between physiological biomarkers, creatinine, and lymphocyte percent in particular, with DNA methylation-based biological aging, suggesting potentially prominent role of kidney and immunity functions on accelerating biological aging. In contrast, alkaline phosphatase showed little correlation with biological aging measures.

Common biomarkers used in aging investigations include telomere length, physiological measures, and epigenetic clocks [4, 47, 48]. At the molecular level, biological aging changes have been translated to several domains including telomere attrition, epigenetic alterations, genomic instability, mitochondrial dysfunction, cellular senescence, stem cell exhaustion, and altered intercellular communication [4, 49]. An important goal of measuring aging is to identify both modifiable targets and informative surrogate endpoints that can be used to track effects of aging interventions in humans [50, 51]. There are several qualities that have to be recognized in aging measures to allow optimal function and replication [50]. Reliability, sensitivity to changes in aging, and feasibility in terms of access and costs are among the most important qualities that make an aging metric widely implemented and accordingly optimize its development and generalizability [50, 52]. Existing evidence supports associations between telomere length and global DNA methylation in youth, which are suggested to affect genome stability and disease risk susceptability [53]. In addition, DNA methylation-based estimator of telomere length is suggested to be more strongly related to age compared to measured telomere length [54].

Saliva-based biomarkers are likely to provide real-time reflections of the individual’s health at the time of collection [14]. In addition to their highly economic advantages and accessibility, saliva is generally a preferred means for populations and age groups among whom a blood draw is not accessible and in clinically difficult situations [6]. The ease of access however should be considered alongside factors that directly affect the composition and molecular abundance in saliva tissue. Saliva is secreted in response to sympathetic and parasympathetic stimuli, and nervous stimuli variations directly affect saliva characteristics in terms of volume, viscosity, protein, and mucin concentrations. There are suggestions that telomere length may not be the ultimate biological aging measure; in addition, qPCR may be less optimal compared to other methods [3, 55, 56]. For DNA methylation measured in saliva, recent evidence suggests that variations seen across cell types, compared to within a cell type, are likely due to variations in immune cell contamination [33, 57]. Moreover, saliva composition could be affected by medications use, age, circadian rhythms among other individual-specific exposures, and conditions such as diabetes, multiple sclerosis, liver conditions, and infectious diseases [6, 58, 59]. Therefore, it is important to consider tissue type and method of measurement in biological aging investigations. Recent data have also shown the utility of measuring DNA methylation in saliva and buccal cells with amounts as low as 10 ng of genomic DNA producing reproducible results [60–62].

In terms of blood-based biomarkers, in the present study, we used DNA methylation and physiology-based measures of aging biology. DNA methylation is the mechanism by which a methyl (CH3) group is added to DNA resulting in modification of genetic function without changes to DNA sequence. This process regulates gene expression and therefore plays an important role in human development and disease [63]. DNA methylation is suggested to be both tissue and disease specific. Different tissue types have been observed to show strikingly varying epigenetic profiles [64]. Commonly used tissues include blood and skin as illustrated in the present investigation [25]. In addition, differences in DNA methylation are suggested to be also tissue-state specific. For example, genome-wide analysis of DNA methylation in the right coronary artery has shown differences in DNA methylation patterns in areas with advanced atherosclerosis compared to areas that are atherosclerotic resistant and the great saphenous vein obtained from same patients [65]. Tissue-based differences in DNA methylation could provide opportunities for novel therapeutic targets and tissue areas related to variations in response to treatment [64, 65]. However, DNA methylation remains to be challenged by cost-related barriers and limited consistency in the measurements as there is no gold standard to define the optimal genome sites and methods of measurements in humans [66, 67]. Therefore, comprehensive comparisons provide a rigorous and reproducible way to capture biological aging effects. Prior evidence suggests that a substantial proportion of data points do not yield the equivalent value when re-quantified from the same DNA sample; furthermore, repeated measures are crucial to uncover consistent replicable signals of DNA methylation dynamics across important variables including time, between populations, and between exposures [68]. That said, cross-tissue variability in DNA methylation profiles has been suggested to be more concordant across tissues than gene expression changes across tissues with age [21, 28]. Physiology-based measures largely represent composites of blood-based analytes that represent the various body domains that change with aging [1]. In the present study, we opted to use phenotypic age, representing parameters that have been validated as predictors of aging in comparable settings including albumin, creatinine, glucose, [log] C-reactive protein [CRP], lymphocyte percent, mean cell volume, red blood cell distribution width, alkaline phosphatase, and white blood cell count [34]. A core motivation to using phenotypes of physiological biomarkers is that one biomarker might not be sufficient to delineate the underlying pathogenesis; therefore, combinations of biomarkers provide an added value over single biomarkers and hence more powerful diagnostic and prognostic tools [69, 70].

The wide variations in the relationships between each physiology biomarker individually with DNA methylation-based biological aging measures suggest differences in how each body system represented by physiology biomarkers interacts with biological aging at the DNA methylation level. For example, creatinine, a biomarker of kidney function, and lymphocyte percent, a blood and immunity biomarker, showed strong positive and negative correlations respectively with some of the DNA methylation-based biological aging measures that exceeded their correlation with chronological age in the same sample [71–75]. These strong correlations suggest potent role of the renal and immunity systems in accelerating biological aging.

There are several limitations in the present study. The biomarkers were measured at two different time points. This time difference could potentially introduce imprecision through impacting the aging profiles across time and power; however, time differences were adjusted for across all the models. In addition, measures of biological aging other than telomere length were not available in saliva; however, the sample was restricted to individuals with complete information for the three measures and therefore allowed for within-individual comparisons. Moreover, biological aging measures are likely complementary to each other rather than alternative exclusive tools since they capture different aspects of the aging process [4, 49]. Our study was also limited in the number of measurements on each individual; therefore, we were not able to assess changes in biomarkers over a lengthy period of time. Repeated measures however would mainly serve as a confirmatory step and might not be feasible to assess over long durations for the same population. Lastly, age ranged between 59 and 72 in the present investigation, and therefore, the results cannot be extrapolated to other age groups and younger individuals who joined HRS after 2008 [15].

Collectively, differences in methods of measurement, cost, accessibility, and generalizability between saliva and blood-based measures determine their applicability in large-scale investigations and the feasibility of their integration in clinical practice [14, 76, 77]. It is also important to consider that blood and saliva secretions are interconnected in many ways, presumably a drug circulating in blood passes through capillary wall, the basement membrane, and glandular epithelial cells prior to being secreted into the salivary duct. This plasma-salivary interchange is bound by active and passive processes and an ultrafiltration step. Drug- or compound-specific characteristics also play a role in this process [6, 14]. Therefore, uncovering connections between both tissues represent an important step towards optimization of accessibility for integration in clinical practice and wide-scale investigations.

## Conclusions

Results of the present investigation suggest strong connections between aging biology biomarkers in saliva and blood in older adults. We observed variations in the relationship between each body system represented by physiology biomarkers and biological aging, particularly at the DNA methylation level. These observations could provide novel opportunities for integration of both blood-based and saliva-based biomarkers in clinical care of vulnerable and clinically difficult to reach populations where either or both tissues would be accessible for clinical monitoring purposes.

## Supplementary Material

Supplementary Material

## Figures and Tables

**Table 1:** Detailed sample characteristics

Characteristic	Summary (unweighted)
*N*	2406
Chronological age (years), median (IQR)^a^	66 (59, 72)
Female, %	1449 (60)
Time difference, median (IQR)^b^	8 (8, 8.3)
Race	
African American, *N* (%)	270 (11)
Self-reported health	
Excellent	350 (15)
Very good	815 (34)
Good	732 (31)
Fair	359 (15)
Poor	98 (4)
**Biomarkers** ^c^	
Saliva based	
Telomere length, median (IQR)	1.30 (1.14, 1.50)
Blood-based physiological measure	
Phenotypic age, median (IQR)	74.41 (65.6, 83.55)
Blood-based DNA methylation measures^d^	
Chronological age (years), median (IQR)^e^	74 (67, 80)
Horvath, median (IQR)	69.00 (63.00, 75.00)
Hannum, median (IQR)	57.80 (52.06, 63.55)
Levine, median (IQR)	60.70 (54.41, 66.84)
Skin & blood, median (IQR)	73.54 (67.70, 78.25)
Lin, median (IQR)	61.61 (55.09, 68.85)
Weidner, median (IQR)	67.43 (60.90, 76.82)
VidalBralo, median (IQR)	65.15 (61.67, 69.00)
GrimAge, median (IQR)	71.30 (65.64, 77.25)

a Chronological age at time of telomere assessment.

b Time difference represents time years between saliva and blood draw.

c Biological aging measures are scaled per 1-year change;

d *n* = 1, 029 had complete data on salivary telomere length, physiology based, and DNA methylation measures.

e Chronological age at time of blood draw assessment

**Table 2:** Correlation coefficients of the relationship between (1) cross-tissue biomarkers of biological aging and (2) intra-tissue biomarkers of biological aging

Biomarkers (blood based)	Cross-tissue biomarkers		Intra-tissue biomarkers	
	Telomere length (saliva based)		Phenotypic age (blood based)	
	Correlation coefficient	*p*-value	Correlation coefficient	*p*-value
Phenotypic age	−0.0264	0.1959	1.00	–
Horvath	−0.0452	0.1478	0.5776	< 0.001*
Hannum	− 0.0318	0.3082	0.6896	< 0.001*
Levine	− 0.0320	0.3056	0.6525	< 0.001*
Skin & blood	− 0.0367	0.2391	0.6640	< 0.001*
Lin	− 0.0670	0.0317	0.5839	< 0.001*
Weidner	−0.0647	0.0379	0.3229	< 0.001*
VidalBralo	−0.0529	0.0897	0.5204	< 0.001*
GrimAge	−0.0438	0.1608	0.7498	< 0.001*

* Denotes a significant *P*-value with Bonferroni correction [40]

**Table 3:** Cross-tissue comparisons between blood-based biomarkers of biological aging (outcome variable) and (log) saliva-based telomere length (with lowest tertile as reference)

Biomarkers^a^	β	95% *CI*	*p*-value
Physiological measure, phenotypic age	–		
2nd tertile	− 2.30	− 4.30, − 0.30	0.024
3rd tertile	− 2.38	− 4.37, − 0.38	0.019
Horvath			
2nd tertile	− 1.10	− 3.27, 1.07	0.320
3rd tertile	− 1.55	− 3.74, 0.63	0.163
Hannum			
2nd tertile	− 1.61	− 3.59, 0.36	0.109
3rd tertile	− 2.40	− 4.39, − 0.40	0.018
Levine			
2nd tertile	− 1.10	− 3.35, 1.14	0.335
3rd tertile	− 2.45	− 4.71, − 0.19	0.033
Skin & blood			
2nd tertile	− 0.99	− 2.88, 0.88	0.298
3rd tertile	− 1.91	− 3.81, − 0.02	0.047
Lin			
2nd tertile	0.12	− 2.40, 2.65	0.921
3rd tertile	− 3.45	− 6.00, − 0.91	0.008
Weidner			
2nd tertile	− 1.27	− 4.05, 1.50	0.369
3rd tertile	− 3.97	− 6.76, − 1.17	0.005
VidalBralo			
2nd tertile	− 1.49	− 2.93, − 0.06	0.040
3rd tertile	− 2.11	− 3.55, − 0.67	0.004
GrimAge			
2nd tertile	− 1.75	− 3.56, 0.05	0.057
3rd tertile	− 3.33	− 5.15, − 1.51	< 0.001

a Measures per tertile increase in telomere length with lowest tertile as reference; models were adjusted for sex, sex and telomere length interaction, and time difference between saliva and blood draw
